# Social Vulnerability Factors and Reported Post-Disaster Needs in the Aftermath of Hurricane Florence

**DOI:** 10.1007/s13753-020-00315-5

**Published:** 2020-11-03

**Authors:** Julia Crowley

**Affiliations:** Department of Criminology and Criminal Justice, Western Carolina University, Cullowhee, NC 28723 USA

**Keywords:** FEMA, Hurricane Florence, Social vulnerability, Post-disaster needs

## Abstract

This research examines the relationship between social vulnerability factors and reported needs following Hurricane Florence. Weighted least squares regression models were used to identify predictor variables for valid registrations that reported needs pertaining to emergencies, food, and shelter. Data consisted of zip codes in North Carolina and South Carolina that received individual assistance for Hurricane Florence (*N *= 406). The results suggest that when controlling for event-specific factors and flood mitigation factors, the proportions of the population that is female, the population over 65, the population aged 5 and under, the population older than 5 years not speaking English, and the minority population were all predictors of the per capita reported emergency needs. When controlling for the same variables, the proportions of the population over the age of 25 with a Bachelor’s degree, the female population, the population aged 5 and under, the population above 5 years old that does not speak English, and the minority population were all predictors of the per capita reported food needs. With the same variables controlled for, three variables—the proportions of the population over 65, the population aged 5 and under, and the non-English-speaking population above 5 years of age—were all predictors of the per capita reported shelter needs. The results suggest that more attention should be given to these vulnerable populations in the pre-disaster planning process.

## Introduction

Disaster events are global occurrences, but the individual impacts are often influenced by vulnerability. The United Nations International Strategy for Disaster Reduction (UNISDR) defines vulnerability as “the characteristics and circumstances of a community, system or asset that make it susceptible to the damaging effects of a hazard” (UNISDR 2009, p. 30). More specifically, social vulnerability plays an important role in the outcomes of community and individual post-disaster needs. Cutter et al. (2003) describe social vulnerability as a combination of social inequalities and place inequalities. Social inequalities refer to the social factors that influence susceptibility to harm and the ability to respond, while place inequalities are characteristics of communities and the built environment. Several studies have examined the relationship between social vulnerability and resilience (Gall 2013; Bergstrand et al. 2015; Pollnac et al. 2015; Joakim et al. 2016; Ran et al. 2020). According to Cutter et al. (2008, p. 599), “resilience is the ability of a social system to respond and recover from disasters and includes those inherent conditions that allow the system to absorb impacts and cope with an event, as well as post-event, adaptive processes that facilitate the ability of the social system to re-organize, change, and learn in response to a threat.” Bergstrand et al. (2015) found a correlation between high levels of vulnerability and low levels of resilience among U.S. counties. Lightfoot et al. (2020) frequently observed an ongoing, chronic anxiety regarding health and family among women in the post-disaster environment. They conclude that this may inhibit personal resilience and the ability to cope with future events.

Other studies cite gender inequality as a characteristic of social vulnerability and its impacts on disaster response and recovery. For example, Fothergill (1998) argued that women’s heightened exposure to hazards is attributed to their social class, their caregiving roles, and their relative lack of power and status. Anastario et al. (2009) examined gender disparities in the context of the disaster recovery process following Hurricane Katrina. They found that the rate of gender-based violence among internally displaced people in traveler trailer parks in Mississippi increased within the year following Katrina, and did not return to baseline during the protracted phase of displacement. Haney and Gray-Scholz (2020) indicate that women are more likely to experience disrupted perceptions of order and security in the post-disaster environment, and Neumayer and Plumper (2007) assert that natural hazards and disasters lower the life expectancy of women more than that of men. In addition, Akerkar and Fordham (2017) conclude that gender differences in disasters are ubiquitous globally, even in societies that appear to have gender equality.

Income is another factor that is commonly described in the literature as a component of social vulnerability. Fang et al. (2017) find that variation in the wealth of individual U.S. counties alone explained 12.4% of the impacts of natural hazard-induced disasters between counties. On a similar note, Kahn (2005) concludes that the average quantities of post-disaster deaths, injuries, and homelessness are reduced as income increases. Furthermore, Peacock and Girard (1997) assert that economically and socially disadvantaged individuals are more likely to reside in housing that is substandard and are at a greater risk of being damaged from a disaster event. Hamideh and Rongerude (2018) cite income as a determinant of an individual’s level of participation in post-disaster recovery decision making. Building off of that insight, Ogie and Pradhan (2019) describe the multiple dimensions of social vulnerability that are influenced by the income factor. For example, low-income individuals who cannot afford vehicles may be trapped during a disaster if public transportation or emergency mass transit is not available. Sadowski and Sutter (2005) offer a conflicting explanation of the role of income, however, and suggest that an increase in income will increase coastal populations, which in turn increases hurricane damage.

An additional social vulnerability factor that has been highlighted in the literature involves the disproportionate impacts of disaster events on different age groups. Very young children exhibit unique challenges and needs in the post-disaster environment. Wilson and Kershaw (2008) surveyed childcare personnel in 14 Florida counties. Responses demonstrated a need and desire for greater support in regards to disaster preparedness, and an increased availability for training sessions on the emotional needs of children following disaster events. Callaghan et al. (2007) estimated that 75,000 infants were directly affected by Hurricane Katrina. These authors explain that exposure to environmental contaminants, psychological stress, and a lack of access to healthcare and medications during a disaster event can potentially pose serious consequences for infants. Elderly populations are also classified as a vulnerable group in the literature. For example, Malik et al. (2018) observed that older adults present higher risks for worse health outcomes with increased emergency department visits following disasters. Similarly, Har (2016) asserts that older adults have increased morbidity and mortality in the post-disaster environment due to factors like preexisting physical and mental illnesses, disabilities, and specific care needs at an individual level. In contrast, Rafiey et al. (2016) found that there is an overall higher level of positive mental health among elderly earthquake survivors in Iran compared to their younger counterparts. Daddoust et al. (2018) conclude that the level of social vulnerability of the elderly to disasters is highly influenced by other factors, which include individual characteristics, economic conditions, culture, and residence.

An individual’s educational attainment is another social vulnerability factor that is frequently cited throughout the literature. Land and Hummel (2013) analyzed two regions in the West African Sahel that have been heavily impacted by climate change and environmental degradation. They found that formal education plays an important role in reducing vulnerability to environmental stress because individuals with higher education levels tend to be less dependent on environmentally sensitive economic activities such as farming. On a similar note, Chen et al. (2013) argue that more education translates to a greater capacity to respond to, cope with, and recover from natural hazard-induced disasters. In addition, Ribeiro et al. (2018) studied the impacts of education on the health of a socially vulnerable population in Brazil. They found that social policies that reduced the illiteracy rate and improved accessibility to schools resulted in positive impacts on the health of local residents.

The literature also describes linguistic minorities as a factor influencing social vulnerability to disaster events. Uekusa (2019) analyzed the complex experiences of linguistic minorities in the 2010–2011 Canterbury and Tohoku disaster events, and found that these groups confront unique disaster vulnerabilities, partly due to language barriers. Teo et al. (2019) surveyed 180 residents from a variety of ethnic and language backgrounds in Logan City, Australia, and found that policymakers need to give more consideration to how different ethnic groups understand and prepare for disasters. They also recommend that policymakers design disaster management and communication plans that provide the needed language translations.

An additional social vulnerability factor that is frequently cited in the literature consists of race and ethnicity. Aksha et al. (2019) found there to be particularly high levels of social vulnerability in areas of Nepal that have high concentrations of minority populations. Furthermore, de Loyola Hummell and colleagues examined social vulnerability in Brazil, and observed that larger cities with more diverse racial concentrations tend to exhibit more disparities in income and education, which in turn contributes to increased social vulnerability (Loyola Hummell et al. 2016). Disparities in the levels of social vulnerability among particular racial and ethnic groups have also been highlighted in studies that were conducted in the United States. For example, Spence et al. (2007) surveyed 935 Katrina evacuees, and found differences in crisis preparation and information seeking on the basis of race. In addition, James et al. (2007) concluded that there is a lack of culturally appropriate emergency risk communication for low-income minorities. Racial disparities have also been highlighted in the impacts of COVID-19. Kim and Bostwick (2020) found that the proportion of African American residents in Chicago communities has an independent effect on the COVID-19 death rate.

A substantial body of literature also examines the willingness of individuals to request disaster assistance. This literature addresses the role of social vulnerability, particularly as it pertains to the factors that were previously discussed. In the post-disaster environment, Fothergill (1998) suggests that men are more likely to view financial aid as a stigma because the payments challenge their role as the breadwinner. There is also reference to the role of income as a factor influencing one’s willingness to request disaster assistance. Fothergill (2003) conducted a qualitative, longitudinal study of women who survived the 1997 flood in Grand Forks, North Dakota. She observed that many of the participants felt that accepting charity meant that they were no longer members of the middle class. In contrast, Urmson et al. (2016) determined that an individual’s socioeconomic status was not related to their comfort level in seeking disaster assistance. They also hypothesized that older adults may be more likely to develop psychological problems during disasters, making them less likely to seek help. But their results demonstrated that there was no effect of age on an individual’s comfort with seeking help. A study conducted by Rivera (2016) demonstrated a potential negative relationship between an individual’s educational status and whether they were likely to apply for home assistance from the Federal Emergency Management Agency (FEMA). Moreover, Horton (2012) addressed many of the obstacles that are imposed on linguistic, racial, and ethnic minorities when requesting disaster assistance. She cited the disproportionate impacts of the Deepwater Horizon Oil Spill on the Southeast Asian community of the Gulf region, many of whom relied heavily on fishing and seafood processing to make a living. Language barriers proved to be a major deterrent for this group in terms of access to needed disaster assistance. She also described frequent confusion regarding whether deportation laws would be enforced in the aftermath of a disaster. The fear and anxiety this imposes among immigrants—who then become reluctant to ask for government assistance—is considerable.

The purpose of this research is to examine if the social vulnerability factors that were identified in the literature are predictors of reported post-disaster needs following Hurricane Florence. Florence was a Category 1 hurricane that made landfall near Wrightsville Beach, North Carolina on 14 September 2018 (Griffin et al. 2019). Florence has been ranked among the 10 costliest hurricanes in U.S. history with approximately 50% of the damage attributed to uninsured losses caused by residential flooding (Paul et al. 2019). The analysis controls for event-specific factors and factors that can potentially mitigate flood damage. The dependent variables consist of the reported per capita post-disaster needs related to emergencies, food, and shelter. Details on the data collection and analysis are provided in the next section.

## Materials and Methods

The following section describes the Materials and methods that were employed to conduct the research. This includes the data collection process and sources. The section concludes with a description of the methods that were used to analyze the data.

### Data Collection

The magnitude of Hurricane Florence resulted in major disaster declarations for the states of North Carolina, South Carolina, and Virginia (FEMA 2020a). In the United States, the Robert T. Stafford Disaster Relief and Emergency Assistance Act organizes federal aid to state and local governments that have been devastated by disaster events (FEMA n.d.). Local governments are the first to respond, and if they are overwhelmed, they appeal to their state governments for assistance. The state responds with resources, often deploying the National Guard and teams that conduct damage assessments. If the state needs additional assistance, the Governor can request a major disaster declaration from the federal government. The decision to approve the request is at the discretion of the President. The approval of the major disaster declaration allows for the activation of several federal programs, including assistance from FEMA, in the response and recovery efforts. FEMA disaster assistance falls into three general categories—individual assistance (aid to individuals and households), public assistance (aid to public entities), and hazard mitigation assistance. Hazard mitigation opportunities are assessed in most situations, but some declarations will provide only individual assistance or only public assistance. The determination of program eligibility is based on the needs found during the damage assessment, and any other information that may be discovered at a later time (FEMA n.d.).

For Hurricane Florence, select counties in Virginia were eligible to apply for public assistance only, but in North Carolina and South Carolina, some counties were eligible to apply for both public assistance and individual assistance (FEMA 2020a). The Individuals and Households Program (IHP) is part of individual assistance, and provides money and services to people in the declared areas who have experienced property damage and whose losses are not covered by insurance (FEMA n.d.). The application process for IHP begins with registration by the individual. This may be completed over the phone, online, or at a FEMA Disaster Recovery Center. A valid registration requires that an individual is a resident of the state and county that received a major disaster declaration, and that the registration is completed within the designated FEMA registration period (Grube et al. 2018).

Data related to post hurricane needs through valid registrations for IHP were retrieved from OpenFEMA in the section on Individual Assistance Open Disaster Statistics (FEMA 2020b) on 26 January 2020. The data used for this research consist of 406 zip codes in North Carolina and South Carolina that received major disaster declarations for Hurricane Florence with individual assistance and had valid registrations for the IHP. It is important to note that “FEMA and the Federal Government cannot vouch for the data or analyses derived from these data after the data has been retrieved from the Agency’s website(s) and/or Data.gov” (FEMA 2020b). The previous quote was derived from an excel file that was downloaded from the FEMA website with the title “Individuals and Households Program-Valid Registrations.” The data that were utilized from this source for the impacted zip codes include the valid registrations that reported emergency needs, food needs, and shelter needs. Each of these sources was divided by the zip code populations and was then used as dependent variables to account for specific types of per capita post hurricane reported needs.

Predictor variables were created for each zip code to depict the vulnerable populations that were discussed in the literature review. Each of these variables was also specifically cited in the literature pertaining to the willingness of individuals to request disaster assistance. These include the median household income (US$), the proportion of the population over age 25 with a Bachelor’s degree, the proportion of the population that is female, the proportion of the population over 65, the proportion of the population aged 5 and under, the proportion of the population over 5 years old not speaking English, and the proportion of the population that are minorities. These data were obtained by looking up these characteristics for each individual zip code using SimplyAnalytics (2020). The program derived the datasets through Easy Analytic Software, Inc. (EASI) and the data are based on the Census data that was estimated for 2019 by EASI.

Peak wind gusts (mph) and the distance to the coast (mi) for each zip code were included to control for event-specific factors. These figures were obtained by importing the HURREVAC (Hurricane Evacuation) storm advisory for Hurricane Florence in the impacted regions, and running an analysis for the hurricane model in Hazus. A shapefile of U.S. zip codes was downloaded from Data.Gov (2019) and extracted in ArcMap to only include the zip codes for North Carolina and South Carolina. The extracted zip code shapefile was then added to the Hazus map with the imported HURREVAC storm advisory for Hurricane Florence. After completing the analysis, the Wind Speeds section on the results tab was examined by adding the peak gusts (mph) to the map. The peak gusts were subsequently looked up for each of the 406 zip codes using the Identify tool. The Identify tool also provided information on the distance to the coast for each zip code. The literature suggests that an area’s distance to the coast is an influential predictor of post-hurricane needs. For example, Xian et al. (2018) found that the distance to the coast was a significant determinant of damage in Big Pine Key, Florida in the aftermath of Hurricane Irma. The distance to the coast is also an important predictor for post-Hurricane Florence reported needs given the trajectory of the storm, which made landfall at the eastern coast of North Carolina, and the massive amounts of damage that resulted from flooding and diminished with distance from the coast.

Variables that can potentially mitigate flood damage were also added to the analysis to control for an individual home’s susceptibility to flood damage. These include the proportion of the homeowners with flood insurance and the median home values (US$). While flood insurance cannot prevent damage to homes from hurricanes, it can reduce the repair costs that would otherwise be incurred by the policy holder (Kunreuther 2008; Petrolia et al. 2013). In addition, the literature associates higher home values with higher quality building materials (Fronstin and Holtmann 1994; Sadowski and Sutter 2005; Awondo et al. 2019). Fronstin and Holtmann (1994) assessed the determinants of residential property damage following Hurricane Andrew, and found the assessed value of a given house to be inversely related to the probability of the home being uninhabitable after the storm. Furthermore, Awondo et al. (2019) observed that homebuyers pay an average premium of 7% for homes with windstorm loss mitigation features. These data were obtained by looking up the homeowners with flood insurance and the median home values (US$) for each individual zip code in the analysis using SimplyAnalytics (2020), and the program also derived these datasets through EASI.

### Data Analysis

Descriptive statistics were provided for the variables that pertain to the mean, standard deviation, and minimum and maximum values. Ordinary least squares (OLS) regressions were initially used to examine if the above-mentioned predictor variables were significant predictors of the valid registrations that reported needs pertaining to emergencies, food, and shelter. The assumptions of OLS were then examined. No multicollinearity existed between any of the predictor variables so they were all kept in the models. The Durbin-Watson statistics confirmed that the values of the residuals were independent, and the normal probability plot confirmed that the values of the residuals were normally distributed. Furthermore, the Cook’s Distance for each of the three models did not detect any outliers. But the scatterplot suggests that there was heteroscedasticity. In order to address this, each of the three models was rerun as a weighted least squares (WLS) regression. The results and discussion sections reflect the WLS regressions.

## Results

The following section presents the results of the research. The first subsection discusses the descriptive statistics of the variables. The second subsection provides figures for the results of the weighted least squares regression analyses.

### Descriptive Statistics

Table 1 displays descriptive statistics for the variables, which include the means, standard deviations, minimum, and maximum values. These figures summarize each of the individual variables that were selected for this analysis. The variables in Table 1 are also included in the weighted least squares regression models that are described in Sect. 3.2.

**Table 1:** Descriptive statistics for the variables

Variables (*N* = 406)	Mean	SD	Minimum	Maximum
Per Capita Emergency Needs	0.02	0.03	0.00	0.21
Per Capita Food Needs	0.02	0.02	0.00	0.11
Per Capita Shelter Needs	0.01	0.01	0.00	0.08
Peak Gust (mph)	46.02	46.32	0.00	133.00
Distance to the Coast (mi)	104.61	82.33	0.00	300.00
Proportion of Homeowners with Flood Insurance	0.03	0.00	0.00	0.04
Median Home Value (US$)	138,871.73	83,353.62	0.00	894,345.00
Median Household Income (US$)	58,910.62	19,133.18	0.00	126,933.00
Proportion of the Population with a Bachelor’s Degree (25+)	0.14	0.09	0.00	0.45
Proportion of the Female Population	0.50	0.05	0.00	0.56
Proportion of the Population Over 65	0.16	0.07	0.00	0.46
Proportion of the Population Aged 5 and Under	0.09	0.06	0.00	0.45
Proportion of the Population That Does Not Speak English (5+)	0.14	0.09	0.03	1.00
Proportion of the Minority Population	0.37	0.20	0.01	1.00

### Weighted Least Squares (WLS) Regressions with Per Capita Valid Registrations that Reported Post-Disaster Needs as the Dependent Variables

Table 2 summarizes the coefficients of the predictor variables in the three models with the per capita total valid registrations that reported emergency, food, and shelter needs in the aftermath of Hurricane Florence as the dependent variables. The predictor variables as a group predicted the per capita total valid registrations that reported emergency needs, *F* (11, 394) = 31.259, *p *< 0.001, *f*^2^ = 0.82. This is a strong effect size. The adjusted R-squared value indicates that the variables have 45.1% of their variability in common. The predictor variables as a group also predicted the per capita total valid registrations that reported food needs, *F* (11, 394) = 34.607, *p *< 0.001, *f*^*2*^ = 0.91. This is a strong effect size. The adjusted R-squared value indicates that the variables have 47.7% of their variability in common. Finally, the predictor variables as a group predicted the per capita total valid registrations that reported shelter needs, *F* (11, 394) = 31.099, *p *< 0.001, *f*^*2*^ = 0.82. This is a strong effect size. The adjusted R-squared value indicates that the variables have 45% of their variability in common.

**Table 2:** Weighted least squares (WLS) regressions with valid registrations that reported post-disaster needs as the dependent variables

Dependent variables			
Predictor variables	Per capita emergency needs	Per capita food needs	Per capita shelter needs
Constant	0.022** (0.006)	0.020** (0.007)	0.005* (0.002)
Peak Gust (mph)	2.877E−5** (0.000)	3.189E−5 *** (0.000)	1.664E−5** (0.000)
Distance to the Coast (mi)	− 8.300E−5*** (0.000)	− 6.206E−5*** (0.000)	− 2.795E−5*** (0.000)
Proportion of Homeowners with Flood Insurance	− 1.019*** (0.284)	− 0.768* (0.320)	− 0.118 (0.103)
Median Home Value (US$)	− 1.527E−8** (0.000)	− 1.027E−8* (0.000)	4.334E−9 (0.000)
Median Household Income (US$)	7.261E−9 (0.000)	5.071E−8 (0.000)	− 2.391E−9 (0.000)
Proportion of the Population with a Bachelor’s Degree (25+)	− 0.001 (0.007)	− 0.013** (0.005)	− 0.001 (0.002)
Proportion of the female population	0.071*** (0.010)	0.047*** (0.007)	0.001 (0.004)
Proportion of the population over 65	0.023* (0.010)	0.007 (0.008)	0.033*** (0.006)
Proportion of the population aged 5 and under	0.048** (0.015)	0.028* (0.011)	0.034*** (0.007)
Proportion of the population that does not Speak English (5+)	− 0.038*** (0.004)	− 0.026*** (0.004)	− 0.011*** (0.002)
Proportion of the minority population	− 0.008** (0.003)	− 0.005* (0.002)	0.000 (0.001)
Adjusted R-Squared	0.451	0.477	0.450
F-Statistic	31.259***	34.607***	31.099***
Durbin-Watson Statistic	1.887	1.918	1.828
*N*	406	406	406

Standard errors are reported in parentheses

*p value < 0.05

**p-value < 0.01

***p-value < 0.001

The peak gusts, distance to the coast, homeowners with flood insurance, median home value, female population, population over 65, population aged 5 and under, population over age 5 that does not speak English, and the minority population were all predictors of the per capita valid registrations that reported emergency needs following Hurricane Florence. As the peak gusts increased, the reported per capita emergency needs increased (*B *= 2.877E−5, *p *= 0.005), and as the distance to the coast decreased, the reported per capita emergency needs increased (*B *= − 8.300E−5, *p *< 0.001). In addition, as the homeowners with flood insurance decreased, the reported emergency needs increased (*B *= − 1.019, *p *< 0.001), and as the median home value decreased, the reported emergency needs increased (*B *= − 1.527E−8, *p *= 0.006). In terms of the social vulnerability variables, the female population, population over 65, and the population aged 5 and under were positive predictors of the per capita reported emergency needs such that the per capita reported emergency needs increased by 0.071 for each percent of the female population (*B *= 0.071, *p *< 0.001); the per capita reported emergency needs increased by 0.023 for each percent of the population over 65 (*B *= 0.023, *p *< 0.020); and the per capita reported emergency needs increased by 0.048 for each percent of the population aged 5 and under (*B *= 0.048, *p *= 0.001). The population over age 5 that does not speak English and the minority population were negative predictors of the per capita reported emergency needs such that the per capita reported emergency needs decreased by 0.038 for each percent of the population age 5 and over not speaking English (*B *= − 0.038, *p *< 0.001); and the per capita reported emergency needs decreased by 0.008 for each percent of the minority population (*B *= − 0.008, *p *= 0.001). The median household income and the population older than 25 with a Bachelor’s degree were not predictors of the per capita valid registrations that reported emergency needs.

The control variable outcomes for the per capita valid registrations that reported food needs following Hurricane Florence were similar to those of the per capita reported emergency needs as the peak gusts, distance to the coast, homeowners with flood insurance, and the median home value were all predictors. As the peak gusts increased, the per capita reported food needs increased (*B *= 3.189E−5, *p *< 0.001); as the distance to the coast decreased, the per capita reported food needs increased (*B *= − 6.206E−5, *p *< 0.001); as the homeowners with flood insurance decreased, the per capita reported food needs increased (*B* =− 0.768, *p *= 0.017); and as the median home value decreased, the per capita food needs increased (*B *= − 1.027E−8, *p *= 0.023). The social vulnerability variables that were statistically significant predictors of the per capita reported food needs include the population over 25 with a Bachelor’s degree, the female population, the population aged 5 and under, the population older than 5 that does not speak English, and the minority population. The female population and the population component aged 5 and under were positive predictors of the per capita reported food needs such that the per capita reported food needs increased by 0.047 for each percent of the female population (*B *= 0.047, *p *< 0.001); and the per capita reported food needs increased by 0.028 for each percent of the population aged 5 and under (*B *= 0.028, *p *= 0.015). The population older than 25 with a Bachelor’s degree, the population older than 5 that does not speak English, and the minority population were negative predictors of the per capita food needs such that the per capita reported food needs decreased by 0.013 for each percent of the population over 25 with a Bachelor’s degree (*B *= − 0.013, *p *= 0.005); the per capita reported food needs decreased by 0.026 for each percent of the population above 5 years old that does not speak English (*B *= − 0.026, *p *< 0.001); and the per capita reported food needs decreased by 0.005 for each percent of the minority population (*B *= − 0.005, *p *= 0.013). The median household income and the population over 65 were not statistically significant predictors of the per capita reported food needs.

The third model with per capita valid registrations that reported shelter needs as the dependent variable differed from the previous two models in terms of the control variables. The peak gusts and the distance to the coast predicted the per capita shelter needs such that as the peak gusts increased, the per capita reported shelter needs increased (*B *= 1.664E−5, *p *= 0.004), and as the distance to the coast decreased, the per capita reported shelter needs increased (*B *= − 2.795E−5, *p *< 0.001). However, the homeowners with flood insurance and the median home value were not statistically significant predictors of the per capita reported shelter needs. The age variables were positive predictors of per capita reported shelter needs such that the per capita reported shelter needs increased by 0.033 for each percent of the population over 65 (*B *= 0.033, *p *< 0.001); and the per capita reported shelter needs increased by 0.034 for each percent of the population aged 5 and under (*B *= 0.034, *p *< 0.001). The population over 5 years old that does not speak English was the only negative predictor of per capita reported shelter needs such that the per capita reported shelter needs decreased by 0.011 for each percent of the population older than 5 years not speaking English (*B *= − 0.011, *p *< 0.001). The median household income, the population older than 25 with a Bachelor’s degree, the female population, and the minority population were not statistically significant predictors of the per capita reported shelter needs.

## Discussion

The statistical insignificance of the median household income variable as a predictor in all three models correlates with the conflicting views that were depicted in the literature on the role of income in relation to social vulnerability following a disaster event. On the one hand, lower-income residents of the impacted zip codes might have been more inclined to report post-disaster needs due to a lack of resources, but it is also possible that their low-income status prevented them from acquiring material items like cell phones and vehicles (Ogie and Pradhan 2019) that might be required to take the necessary steps to report post-disaster needs. In addition, lower-income individuals are more susceptible to hurricane damage because they are more likely to live in substandard housing (Peacock and Girard 1997), but higher-income individuals are more likely to live in coastal areas (Sadowski and Sutter 2005), which also increases their vulnerability. It is also likely that income had different influences on an individual’s decision to request disaster assistance based on their own values and perceptions. For example, some middle-class residents of the impacted zip codes might have viewed the need for disaster assistance as a threat to their middle-class status (Fothergill 2003), while other middle-class residents might have had no issues with making such a request (Urmson et al. 2016). In sum, the role of income as a predictor of reported post-disaster needs is complicated because the variable consists of many different facets that can influence one’s decision or ability to request such assistance.

As a negative predictor of the reported post-disaster food needs, the over 25 year old population with a Bachelor’s degree corresponds with previous studies that suggest requests for disaster assistance decreases as education increases (Rivera 2016). Although it is surprising that education is not a statistically significant predictor of reported post-disaster emergency and shelter needs, it is possible that an increased education creates more opportunities for individuals to adopt practices that promote food security (Keenan et al. 2001; Hamm and Bellows 2003).

Previous studies support the finding of the female population as a statistically significant, positive predictor of reported post-disaster emergency and food needs. It is possible that more women than men reported these particular needs after Florence due to increased female anxieties over the health and well-being of family members (Lightfoot et al. 2020), disproportionately higher caregiving responsibilities compared to male household members, and an overall lower status in society (Fothergill 1998). An additional explanation could be the increased likelihood for men to view the disaster assistance as a threat to their role as the breadwinner (Fothergill 1998). The lack of statistical significance of the female population as a predictor of reported post-disaster shelter needs could be the result of conflicting gender-based views between shelter needs and a disproportionately higher apprehension felt by women when compared to men about accepting accommodations in places they are not familiar with due to safety concerns.

The overall results for the age-related variables also appear to correspond with those of previous studies. The population over 65 as a statistically significant, positive predictor of reported post-disaster emergency needs and shelter needs could be explained by their overall higher risk for preexisting health conditions (Har 2016) and emergency department visits following a disaster event (Malik et al. 2018). These results also support the findings of Urmson et al. (2016) that age was not a hindrance towards an individual’s comfort with seeking help. The statistical insignificance of the population over 65 as a predictor of reported post-disaster food needs could be the result of conflicting individual scenarios where some members of the population over 65 have disproportionately higher food needs, while others have access to federal food and nutrition programs (Gergerich et al. 2015). Furthermore, the statistical significance of the population aged 5 and under as a positive predictor of the dependent variables in all three models could be the result of specific challenges faced by this population when needed resources and routines are disrupted by a disaster event (Callaghan et al. 2007). Such challenges likely generate needs that resulted in the parents or guardians applying for disaster assistance.

It is alarming to observe that the proportion of the population over age 5 that does not speak English is a statistically significant negative predictor of the dependent variables for all three models. This corresponds with the findings of Horton (2012) regarding language barriers as a hindrance to requesting disaster assistance. It is possible that linguistic minorities reported less post-disaster need overall because they could not find anyone to translate the guidelines for requesting assistance.

The minority population as a statistically significant negative predictor of reported post-disaster emergency needs and food needs contradicts previous studies that suggest that minority populations tend to have disproportionately higher needs in the aftermath of a disaster (Loyola Hummell 2016; Aksha et al. 2019). These findings can be attributed to a lack of culturally appropriate emergency risk communication for minorities (James et al. 2007), concerns regarding deportation (Horton 2012), and a general lack of trust in the government.

## Conclusion

The overall results of this research indicate that when controlling for peak gusts (mph), the distance to the coast (mi), the proportion of homeowners with flood insurance, and median home value (US$), the proportion of the population that is female, the proportion of the population over 65, the proportion of the population aged 5 and under, the proportion of the population over 5 years old not speaking English, and the proportion of the population that are minorities were all predictors of the per capita reported emergency needs following Hurricane Florence. When controlling for the same variables, the proportion of the population older than 25 with a Bachelor’s degree, the proportion of the population that is female, the proportion of the population aged 5 and under, the proportion of the population over 5 years old that does not speak English, and the proportion of the population that are minorities were all predictors of the per capita reported post-disaster food needs. Finally, when also controlling for the same variables, the proportion of the population over 65, the proportion of the population aged 5 and under, and the proportion of the population over 5 years of age not speaking English were all predictors of the per capita reported post-disaster shelter needs.

These results highlight disproportionate post-hurricane needs for specific community characteristics, and suggest that more emphasis should be placed on these groups when planning for disaster response and recovery operations. One limitation of this research is that it does not account for vulnerable populations who did not report needs due to an inability to register. It is possible that a disproportionately higher number of groups from vulnerable populations had specific post-hurricane needs, but did not register as a result of accessibility issues, a lack of knowledge about the registration process, or a variety of additional reasons. Further research is needed on this topic.

Another limitation of this research pertains to the lack of a variable that measures social capital. Variations of post-hurricane emergency, food, and shelter needs can be heavily influenced by community social capital. Further research should explore this variable at the zip code level.

Since data used in this study consist solely of self-reported needs at registration and not the total amounts of damage that was observed by FEMA inspectors and the total amount of Individuals and Households Program (IHP) assistance that was awarded, it is not possible to quantify the value of each individual need. Further research is needed to compare these results to data that represent assessed damages and the amount of IHP funding.

An additional limitation of this research is related to the possibility of errors in the data since the registrations came from different areas and sources. These sources include the FEMA registration phoneline and website as well as the FEMA Disaster Recovery Centers. It is possible that there were inaccuracies in the files due to errors that were made when combining the data.

Nonetheless, this research provides an important contribution pertaining to requests for post-disaster needs by highlighting specific vulnerable groups that exhibited fewer requests for assistance following Hurricane Florence. It is recommended that local emergency management agencies reach out to these groups in advance. Another suggestion involves including post-disaster assistance request processes as a component in local emergency response plans.
